# The Ward Laboratory versus the Institutional Laboratory

**Published:** 1912-06-01

**Authors:** 


### THE MAKING OF A MODERN HOSPITAL.*

### The Ward Laboratory versus the Institutional Laboratory.

It may reasonably be asked, Why cannot all investigations be carried on in the large general laboratories attached to every well-equipped modern hospital—the chemical in the chemical laboratories, the bacteriological in the bacteriological department, and the pathological in the pathological department? Why is it necessary to have a separate and small ward laboratory? Does not this latter method entail a duplication of energy and force, an increase of expense, and a waste of material?

The answer is, that to send bedside specimens to the general laboratories means, in general, a waste of valuable time. If the certainty on which the diagnosis or the continuance of a definite method of treatment depends can be gained by an immediate examination at the bedside, or at least in the wards, it is obviously of the greatest importance that it should be sought for without loss of time. Generally a brief, but necessarily careful and expert investigation in the ward laboratory suffices to clinch the diagnosis or to give the physician or surgeon that information that enables him to go on with or to abrogate his previous line of treatment. Further conclusive data are to be gained from methodical investigations conducted in the general laboratories; in some cases, for instance, where the certainty is only to be gained after bacteriological culture and inoculation experiments have been made, such further investigations, owing to their complicated nature and to the fact that they entail special apparatus and conditions which cannot be obtained in the ward laboratory, must be conducted in the general laboratories, but in the main the preliminary examinations are best done on the spot.

The ward laboratory is therefore a very necessary adjunct to the general laboratories, to which it is, as it were, a sort of clearing-house or advance dépôt. By eliminating the easier examinations, it enables the physician to send to the larger laboratories only those specimens which need a more extended and complicated investigation. By enabling the easier examinations to be done on the spot, without loss of time, it gives him the means of satisfying himself that his diagnosis and treatment are correct. In that way the ward laboratory is an indispensable constituent of the ward unit. The fact that it has of late years become necessary to make it more complete, and to equip it with apparatus which in some cases is costly and delicate, has tended to increase the cost of the ward unit itself, and therefore, as a necessary consequence, to raise the cost per bed. When it was possible, as it formerly was, to conduct all this clinical ward work on the window-sill or on a small ward-table, by means merely of a spirit-lamp, a set of reagents and a test-tube, and a simple microscope, the cost of such a ward laboratory was relatively small. Nowadays, when other apparatus must be provided, and when an expensive, high-power microscope is almost a *sine qua non*, the cost has risen. But at the same time it is a very necessary item of hospital expenditure, and in the interests of the patients in the first, and of the institution in the second place, it is false economy to save on the cost per bed at the expense of the ward laboratory. The importance of careful preliminary examinations conducted in the ward with the least possible delay is so great that a good ward laboratory in connection with every ward unit is imperative.

It must not be thought, however, that by such provision the necessity for a general pathological, bacteriological, or chemical laboratory in connection with the hospital is dispensed with. The ward laboratory, be it clearly understood, serves for the unit; the general laboratory serves for the whole hospital. The large laboratories should be in charge of properly qualified and highly experienced experts who can devote their whole time to the work—often very laborious—which has to be carried on in them. The ward laboratory, on the other hand, exists for the convenience of the medical staff attached to the ward. The investigations that are carried on in it, although they demand for their proper completion expert knowledge and skill, are not of such a highly specialised technique and character as those which are carried on in the larger laboratories. The results obtained are, in general, of immediate value. The ward laboratory is thus an examining station which should be up to date in every respect, and of which the fullest possible use should be made. Where it does not exist, it is justifiable to presume that the institution is not modern in the best sense, in so far that modern methods of diagnosis are not given the prominence which, by virtue of their importance in the treatment of disease, they merit. This is a point on which it is well to lay some stress.

Many hospitals, which spend considerable sums in improving the equipment of their large pathological laboratories, are content to allow such ward laboratories as they have to remain in their primitive state of incompleteness. In such institutions, it will generally be noticed, the staff makes tentative diagnoses while awaiting the findings of researches carried out in the larger laboratories, instead of attempting to gain immediate confirmation by employing bedside methods of pathological investigation which can be carried out at once in the laboratory attached to the ward. The reason for this is mainly the neglect with which this important constituent of the ward unit has hitherto been treated. All the work has been thrown on the larger laboratories with the result that the experts in charge are overwhelmed with material and that a great deal of the preliminary work which should have been carried out in the ward laboratory is delegated to the laboratory boy or to some unqualified assistant.

(*To be continued.*)

* Previous articles appeared on Oct. 7, 14, 21, 28, Nov. 4, 18, Dec. 9, 30, Feb. 10, March 16, April 6, 20, May 4, 18.

